# STIMs and Orai1 regulate cytokine production in spinal astrocytes

**DOI:** 10.1186/s12974-016-0594-7

**Published:** 2016-05-31

**Authors:** Xinghua Gao, Jingsheng Xia, Frances M. Munoz, Melissa T. Manners, Rong Pan, Olimpia Meucci, Yue Dai, Huijuan Hu

**Affiliations:** Department of Pharmacology and Physiology, Drexel University College of Medicine, 245 N. 15th Street, Philadelphia, PA 19102 USA; Department of Pharmacology of Chinese Materia Medica, China Pharmaceutical University, Nanjing, China

**Keywords:** Store-operated calcium channels, STIM1, Orai1, Astrocytes, Cytokine, The spinal cord

## Abstract

**Background:**

Our previous study demonstrated that a store-operated calcium channel (SOCC) inhibitor (YM-58483) has central analgesic effects. However, the cellular and molecular mechanisms of such effects remain to be determined. It is well-known that glial cells play important roles in central sensitization. SOC entry (SOCE) has been implicated in many cell types including cortical astrocytes. However, the role of the SOCC family in the function of astrocytes has not been determined. Here, we thoroughly investigated the expression and the functional significance of SOCCs in spinal astrocytes.

**Methods:**

Primary cultured astrocytes were prepared from neonatal (P2–P3) CD1 mice. Expressions of mRNAs and proteins were respectively assessed by real-time PCR and Western blot analysis. SOCE was measured using a calcium imaging system. Live-cell STIM1 translocation was detected using a confocal microscope. Cytokine levels were measured by the enzyme-linked immunosorbent assay.

**Results:**

We found that the SOCC family is expressed in spinal astrocytes and that depletion of calcium stores from the endoplasmic reticulum by cyclopiazonic acid (CPA) resulted in a large sustained calcium entry, which was blocked by SOCC inhibitors. Using the siRNA knockdown approach, we identified STIM1 and Orai1 as primary components of SOCCs in spinal astrocytes. We also observed thapsigargin (TG)- or CPA-induced puncta formation of STIM1 and Orai1. In addition, activation of SOCCs remarkably promoted TNF-α and IL-6 production in spinal astrocytes, which were greatly attenuated by knockdown of STIM1 or Orai1. Importantly, knockdown of STIM2 and Orai1 dramatically decreased lipopolysaccharide-induced TNF-α and IL-6 production without changing cell viability.

**Conclusions:**

This study presents the first evidence that STIM1, STIM2, and Orai1 mediate SOCE and are involved in cytokine production in spinal astrocytes. Our findings provide the basis for future assessment of SOCCs in pain and other central nervous system disorders associated with abnormal astrocyte activities.

## Background

Glial cells have long been viewed as static constituents of the CNS, primarily serving support functions. This view was dramatically changed when Garrison et al. found that spinal cord astrocytes were activated following nerve injury in 1991 [1]. Accumulating evidence implicates that spinal cord glia are key players in the development and maintenance of pathological pain [2, 3]. There are three major groups of glial cells: astrocytes, microglia, and oligodendrocytes. Astrocytes represent the largest glial cell population in the CNS. Compared with microglia and oligodendrocytes, astrocytes are more closely associated with neurons and blood vessels. One single astrocyte can enwrap four to eight neuronal somata and make a connection with 300–600 neuronal dendrites [4], which allow astrocytes to regulate the external chemical environment of neurons during synaptic transmission. Increasing evidence indicates that microglia play a role in the development of chronic pain conditions, while astrocytes are more important for the maintenance of chronic pain [5]. Notably, several studies showed that bone cancer pain, oxaliplatin- and bortezomib-induced peripheral neuropathy, and collagen-induced arthritis pain models showed marked spinal astrocyte activation with limited microglial activation [6–8]. Although targeting astrocytes is beneficial in treating chronic pain, the inhibition of astrocytes may also attenuate normal astrocyte functions, such as glutamate uptake. Therefore, therapeutic strategies should be directed at more specific astrocyte functions or at specific aspects of reactive astrogliosis, by targeting astrocyte-related molecular mechanisms. However, the molecular mechanisms that regulate specific aspects of reactive astrogliosis such as cytokine production remain elusive.

Store-operated calcium channels (SOCCs) are highly Ca^2+^-selective cation channels that can be activated by depletion of endoplasmic reticulum (ER) calcium stores, as first postulated in 1986 [9]. SOCCs are composed of ER calcium sensors STIM1/2 and pore-forming proteins Orai1/2/3 in most cell types. SOC entry (SOCE) is a major mechanism for triggering Ca^2+^ influx in immune cells. Additionally, SOCE is involved in cytokine production in immune cells [10–12]. Previous studies have demonstrated that YM-58483, a potent inhibitor of SOCCs, blocks SOCCs in immune cells and reduces cytokine production from these cells [13–15]. Our previous study showed that systemic administration of YM-58483 produces strong analgesic and anti-inflammatory actions with peripheral mechanisms associated with reduction of pro-inflammatory cytokines [16, 17]. Interestingly, intrathecal injection of YM-58483 also attenuates pain hypersensitivity [17]. However, the mechanisms underlying its central action remain to be determined. It has also been shown that STIM1 and Orai1 mediate SOCE in cortical astrocytes [18]. It is therefore reasonable to hypothesize that SOCE is an important Ca^2+^ signal in spinal astrocytes. In the present study, we demonstrate that SOCCs are expressed in mouse spinal cord astrocytes. We also identified STIM1, STIM2, and Orai1 as the players of SOCE. Importantly, SOCE can induce TNF-α and IL-6 production, and STIM2 and Orai1 play an important role in lipopolysaccharide (LPS)-induced cytokine production from spinal astrocytes. These findings reveal that SOCE is an important component of Ca^2+^ signaling in spinal astrocytes and regulates cytokine production, suggesting a potential role of SOCCs in central sensitization.

## Methods

### Animals

All experiments were done in accordance with the guidelines of the National Institutes of Health and the Committee for Research and Ethical Issues of IASP and were approved by the Drexel University Animal Care and Use Committee. Pregnant CD1 mice were purchased from Charles River (Wilmington, MA) and individually housed in standard cages in 12-h light/dark cycle.

### Cell culture

Primary cultures of spinal cord astrocytes were prepared from neonatal (P2–P3) mice. Briefly, neonatal mice were decapitated after inducing hypothermia on ice. A laminectomy was performed and the spinal cord was carefully removed. The meninges were removed, and spinal cord strips were then incubated for 30 min at 37 °C in Hank’s balanced salt solution (HBSS) (Invitrogen, Carlsbad, CA) (in mM: 137 NaCl, 5.4 KCl, 0.4 KH_2_PO_4_, 1 CaCl_2_, 0.5 MgCl_2_, 0.4 MgSO_4_, 4.2 NaHCO_3_, 0.3 Na_2_HPO_4_, and 5.6 glucose) containing papain (15 U/ml; Worthington Biochemical, Lakewood, NJ), rinsed three times with HBSS, and placed in minimum essential media (MEM) (Invitrogen) containing 10 % fetal calf serum (FCS, Invitrogen). The strips were mechanically dissociated by gently triturating with a pipette. The resulting cells were plated in 75 cm^2^ flask. Cells were maintained at 37 °C in a humidified atmosphere containing 5 % CO_2_. Culture medium was changed to Dulbecco’s modified Eagle’s medium (DMEM, Invitrogen) containing 10 % FCS after 24 h and then allowed to grow for 6–8 days until 70 % confluent. Cells were shaken in an orbital shaker (320 rpm) for 2 to 3 h to detach cells sitting on top of the astrocyte monolayer (mainly microglia and precursor cells) [19, 20] and then were trypsinized and re-plated to six-well plates for Western blotting analysis, 96-well plates for enzyme-linked immunosorbent assay, or 12-mm coverslips for calcium imaging in MEM culture media.

### Real-time PCR analysis of mRNA expression

Real-time PCR was performed according to our previous study [21]. Total RNA was extracted from astrocytes using TRIzol Reagent (Molecular Research Center, Inc., Cincinnati, OH). The RNA concentration was determined by optical density at 260 nm. Total RNA was reverse transcribed into cDNA for each sample using a Fermentas cDNA synthesis kit (Thermo Scientific, Rockford, IL) following the manufacturer’s instructions. Specific primers for mouse STIM1 (Mm00486423_m1), STIM2 (Mm01223103_m1), Orai1 (Mm00774349_m1), Orai2 (Mm04214089_s1), Orai3 (Mm01612888_m1), and GAPDH were purchased from Applied Biosystems (Foster City, CA). Real-time quantitative PCR was performed in a 7900HT fast real-time PCR System (Applied Biosystems) using the following amplification conditions: 5 min of initial denaturation at 96 °C, then 35 cycles of 96 °C for 30 s, 55 °C for 30 s, and 72 °C for 1.5 min. The threshold cycle for each gene was determined and analyzed using the relative quantitation software (Applied Biosystems). The relative expression of the target genes was calculated using the 2(−Delta Delta C_T,_ 2^−∆∆CT^) method. The mRNA levels of STIM1, STIM2, Orai1, Orai2, and Orai3 were normalized to the housekeeping gene GAPDH.

### Western blot analysis

Astrocytes were washed in PBS and lysed in an ice-cold radio immunoprecipitation assay (RIPA) buffer containing 50 mM Tris HCl, 150 mM NaCl, 0.2 mM EDTA, 1 % Triton X-100, 2 % sodium dodecyl sulfate, 1 % deoxycholate, 0.1 mM phenylmethanesulfonyl fluoride, and protease inhibitor cocktails (Thermo Fisher Scientific). The lysed astrocytes were then sonicated at a constant intensity of 2.5 for 10 s, and centrifuged at 12,000 RPM (4 °C) for 5 min. The concentration of total protein was determined using a Pierce bicinchoninic acid protein assay kit (Thermo Fisher Scientific) following the manufacturer’s instructions. Protein samples were heated at 95 °C for 5 min, electrophoresed in 10 % sodium dodecyl sulfate polyacrylamide gel, and transferred onto polyvinylidene fluoride membranes (Millipore, Billerica, MA). The blots were blocked with 5 % skimmed milk in Tris-buffered saline–Tween 0.1 % for 1 h at room temperature and probed with rabbit anti-STIM1 (1:8000, Cell Signaling, Danvers, MA), anti-STIM2 (1:8000, ProSci, Inc., Poway, CA), anti-Orai1 (1:500, ProSci), anti-Orai2 (1:500, ProSci), anti-Orai3 (1:500, ProSci), and anti-GAPDH (1:10,000, Cell Signaling) primary antibodies at 4 °C overnight. The blots were washed and incubated for 1 h at room temperature with the horseradish peroxidase–conjugated secondary antibody (1:10,000, Cell Signaling) and then developed with enhanced chemiluminescence (Millipore). The densitometry of protein bands was quantified using Image J software (NIH).

### Calcium imaging

Calcium imaging was performed using fura-2-based microfluorimetry and imaging analysis as we previously described [21]. Astrocytes were loaded with 4 μM of fura-2AM (Life Technologies, Grand Island, NY) for 30 min at room temperature in HBSS, washed, and further incubated in normal bath solution (Tyrode’s) containing (in mM) 140 NaCl, 5 KCl, 2 CaCl_2_, 1 MgCl_2_, 10 HEPES, and 5.6 glucose for 20 min. Coverslips were mounted in a small laminar-flow perfusion chamber (Model RC-25, Warner Instruments, Hamden, CT) and continuously perfused at 5 to 7 ml/min with Tyrode’s solution. Images were acquired at 3-s intervals at room temperature (20–22 °C) using an Olympus inverted microscope equipped with a CCD camera (Hamamatsu ORCA-03G, Japan). The fluorescence images were recorded and analyzed using the software MetaFluor 7.7.9 (Molecular Devices). The fluorescence ratio was determined as the fluorescence intensities excited at 340 and 380 nm with background subtraction. Only one recording was made from each coverslip.

### Transfection

For transfection of siRNAs, astrocytes were cultured on glass coverslips (for calcium imaging) and plates (for PCR and Western blot analysis) for 24 h and then transfected with STIM1 siRNA (Dharmacon, Lafayette, CO, USA), STIM2 siRNA, or Scramble siRNA (Dharmacon, Lafayette, CO) using X-tremeGENE HP Transfection Reagent (Roche Applied Science, Indianapolis, USA) following the manufacturer’s instructions as described in our previous study [21]. For transfection of Orai siRNAs, astrocytes were electroporated using a mouse Nucleofector kit according to the manufacturer’s instructions (Lonza Group Ltd, Basel, Switzerland) as described in our previous study [21]. Briefly, astrocytes were transfected with 12 μg of Orai1 siRNA (Life Technologies, Grand Island, NY), Orai2 siRNA (Dharmacon), Orai3 siRNA (Dharmacon), or Scramble siRNA (Life Technologies for Orai1; Dharmacon for Orai2 and Orai3) per 1 × 10^6^ cells. Calcium imaging and Western blot analysis were performed 48–72 h post transfection. For transfection of STIM1-YFP and Orai1-CFP (generous gifts from Dr. Gill, Temple University, PA), astrocytes were electroporated using mouse primary cell Nucleofector kit according to the manufacturer’s instructions (Lonza Group Ltd, Basel, Switzerland). Briefly, astrocytes were transfected with 1.5 μg STIM1-YFP alone or co-transfected with Orai1-CFP (2.5 μg) per 1 × 10^6^ cells and were seeded on 15-mm glass coverslips. After 16 h, transfection medium was removed and astrocytes were fed with fresh culture medium.

### Live-cell confocal imaging

All fluorescence images were captured 48–72 h post transfection using the Olympus FLUOVIEW FV1000 confocal microscope equipped with a ×60 oil-immersion objective. Time-lapse imaging was performed in STIM1-YFP transfected astrocytes. Images of YFP were then acquired at 20-s intervals using a 515-nm laser line for YFP excitation, and YFP emission through a 535- to 565-nm window. For STIM1-YFP and Orai1-CFP co-transfected astrocytes, images of YFP and CFP were acquired at 2-min intervals using 515- and 458-nm laser lines for excitation. All experiments were performed in Tyrode’s solution containing (mM) 140 NaCl, 5 KCl, 1 MgCl_2_, 2 CaCl_2_, 10 HEPES, and 5.6 glucose (pH 7.4) at room temperature. The number of STIM1 puncta was quantified using OLYMPUS FLUOVIEW Ver.3.1b. Regions of interest were randomly selected by drawing 10 by 10 μm square boxes in cell bodies. The same regions were used to count the number of puncta before and after TG or CPA treatment. The puncta were selected as spots of high fluorescence intensity ranging from 0.4 to 2.0 μm in diameter size.

### Enzyme-linked immunosorbent assay

Astrocytes were re-plated in 96-well plates (2 × 10^4^ cells per well). For basal cytokine level measurement, astrocytes were treated with 0.1 % DMSO in MEM culture medium. For measuring cytokine production, astrocyte were first incubated in 0 Ca^2+^ Tyrode’s solution containing (in mM) 140 NaCl, 5 KCl, 1 MgCl_2_, 10 HEPES, and 5.6 glucose for 2 min, then treated with 1 μM TG or 30 μM CPA for 5 min. The 0 Ca^2+^ Tyrode’s solution was replaced with MEM culture medium containing TG for 5 or 24 h or CPA for 24 h. For testing the effect of drugs on TG-induced cytokine production, astrocytes were pretreated with different concentrations of drugs for 15 min before application of TG. Astrocytes were centrifuged at 1000 rpm for 5 min, and IL-6 and TNF-α in the supernatant were measured by enzyme-linked immunosorbent assay (ELISA) according to the manufacturer’s instructions (R&D Systems, Minneapolis, MN, USA).

### MTT cell viability assay

Astrocytes were cultured and re-plated in 96-well plates (1 × 10^4^ cells/well). Astrocytes were first incubated in 0 Ca^2+^ Tyrode’s solution containing (in mM) 140 NaCl, 5 KCl, 1 MgCl_2_, 10 HEPES, and 5.6 glucose for 2 min and then treated with 1 μM TG for 5 min. The 0 Ca^2+^ Tyrode’s solution was replaced with MEM culture medium containing TG for 5 or 24 h. The cell viability was determined by an assay measuring dehydrogenase activity (Trevigen, Gaithersburg, MD) according to the manufacturer’s instructions. Ten microliters of MTT reagent was added to 100 μl of MEM and incubated for 2 h at 37 °C. Once the purple precipitate was clearly visible intracellularly under the microscope, MTT detergent was added to dissolve the formazan crystals. The absorbance was measured directly from 96-well plates at 570 nm using a microplate reader (Spectramax Plus, Molecular Devices, CA).

### Drugs

YM-58483, KN-93, and PD98059 were purchased from Tocris (Minneapolis, MN). Cyclopiazonic acid (CPA), thapsigargin (TG), GdCl_3_, 2-aminoethyl diphenyl borate (2-APB), and LPS were purchased from Sigma (St. Louis, MO). They were dissolved in Milli-Q water or dimethyl sulfoxide (DMSO) as stock solutions and further diluted to final concentrations in 0.1 % DMSO.

### Data analysis

Data are expressed as original traces or as mean ± SEM. Treatment effects were statistically analyzed with a one-way analysis of variance (ANOVA). When ANOVA showed a significant difference, pairwise comparisons between means were performed by the post hoc Bonferroni method. Paired or two-sample Student’s *t* tests were used when comparisons were restricted to two means. Error probabilities of *P* < 0.05 were considered statistically significant. The statistical software Origin 8.1 was used to perform all statistical analyses.

## Results

### SOCCs are expressed in spinal astrocytes

**Fig. 1:**
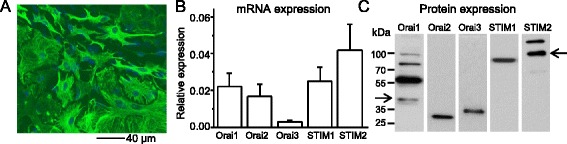
The SOCC family is expressed in spinal astrocytes. **a** Immunostaining of GFAP and DAPI in cultured astrocytes. **b** mRNA levels of STIM1, STIM2, Orai1, Orai2, and Orai3 in cultured astrocytes by RT-PCR (normalized to GAPDH). Values represent mean ± SEM, *n* = 4 samples each. **c** Protein expression of STIM1, STIM2, Orai1, Orai2, and Orai3 in astrocytes by Western blot analysis. *Arrows* indicate the corresponding molecular weights of Orai1 and STIM2 proteins

We previously demonstrated that YM-58483 possesses a strong central analgesic effect in chronic pain conditions [17], suggesting a potential role of SOCCs in central sensitization. We have shown that SOCCs are functional in spinal cord dorsal horn neurons [21]. To examine whether SOCCs are also expressed in spinal cord astrocytes, we first evaluated the purity of astrocytes. Cultured astrocytes were fixed and immunostained with antibodies against glial fibrillary acidic protein (GFAP) (an astroglial marker), Iba1 (a microglial marker), and MAP2b (a neuronal marker). GFAP positive cells represented 92 % of total cells (Fig. 1a), while Iba1 positive cells were either absent or very sparse and MAP2b positive cells were absent (data not shown), indicating that astrocytes are the predominant cell type in our cultured cells. To determine the expression of SOCCs in astrocytes, we performed Taqman RT-PCR and Western blot assays. Figure 1b showed that all mRNAs of the SOCC family were expressed in spinal astrocytes. Western blot results also confirmed the expression of SOCC proteins in spinal astrocytes (Fig. 1c). These results demonstrate that the SOCC family is expressed in spinal astrocytes.

### SOCCs are functional in spinal astrocytes

**Fig. 2:**
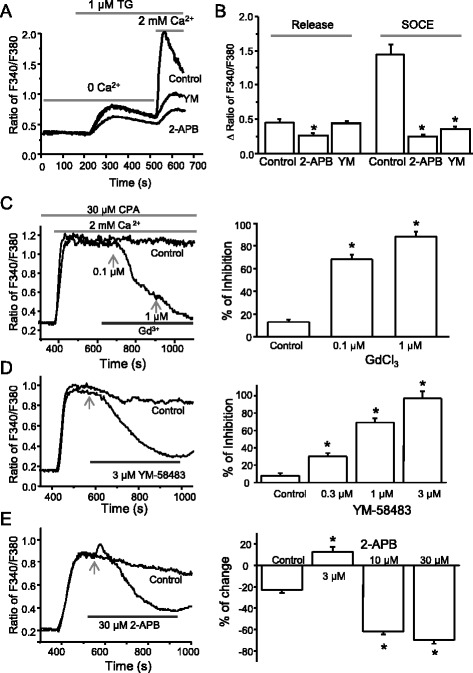
Depletion of endoplasmic reticulum Ca^2+^ stores induces SOC Entry in cultured astrocytes. **a** Representative calcium imaging recordings. **b** Summary of effects of YM-58483 (YM, 3 μM) and 2-APB (30 μM) on TG-induced calcium influx. *n* = 14–22 astrocytes. c-e Effects of GdCl_3_ (**c**), YM-58483 (**d**), and 2-APB (**e**) on CPA-induced calcium entry. *Left panel*, representatives; *right panel*, summary of effects of vehicle (control, *n* = 36), GdCl_3_ (*n* = 19 cells), YM-58483 (*n* = 13–17 cells), and 2-APB (*n* = 12–22 cells) on CPA-induced calcium entry. Values represent mean ± SEM; *P < 0.05, compared with control by the one-way ANOVA

Given the expression of SOCCs in spinal astrocytes, we then asked whether SOCCs are functional in spinal astrocytes. We performed calcium imaging recordings in live astrocytes. When astrocytes were pretreated with the Ca^2+^-free Tyrode’s solution, 1 μM TG transiently elevated intracellular Ca^2+^, and subsequent addition of 2 mM CaCl_2_ caused calcium entry in almost every astrocyte (Fig. 2a). Bath-applied 3 μM YM-58483, an inhibitor of SOCE, did not affect the calcium release induced by TG but dramatically prevented the calcium influx induced by TG (Fig. 2a, b). Another inhibitor of SOCE, 2-APB, significantly reduced both the calcium release and calcium entry of TG (Fig. 2a, b). To test whether these inhibitors attenuate SOCE, astrocytes were pretreated with CPA (another Ca^2+^-ATPase inhibitor) to deplete calcium stores since it produced a more sustained calcium response. Subsequent addition of 2 mM CaCl_2_ induced sustained responses with limited reductions over 10 min. GdCl_3_ completely blocked CPA-induced SOCE at 1 μM concentration (Fig. 2c). YM-58483 markedly attenuated SOCE in a concentration-dependent manner (Fig. 2d). 2-APB slightly increased SOCE at a low concentration and inhibited SOCE at higher concentrations (Fig. 2e). These results indicate that SOCCs are functional in spinal astrocytes.

### Orai1, STIM1, and STIM2 are responsible for SOCE in spinal astrocytes

**Fig. 3:**
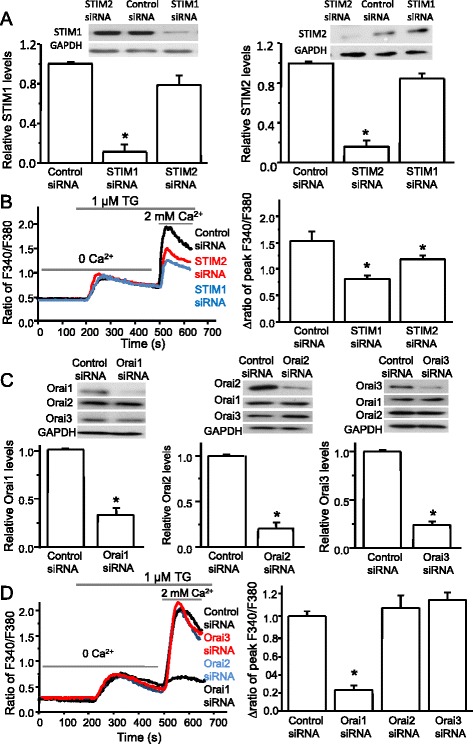
Orai1, STIM1, and STIM2 are important for SOCE in spinal astrocytes. **a** Effects of specific siRNAs against STIM1 or STIM2 on protein levels of STIM1 and STIM2 (*n* = 4–5 samples). **b** Effects of specific siRNAs against STIMs on TG-induced SOCE. *Left panel*, representatives of TG-induced calcium responses recorded in astrocytes transfected with control siRNA or targeting siRNAs against STIMs; *right panel*, summary of effects of control siRNA (*n* = 78), STIM1 siRNA (*n* = 77), and STIM2 siRNA (*n* = 69). **c** Effects of specific siRNAs against Orai1, Orai2, or Orai3 on protein levels of Orai1, Orai2, and Orai3 (*n* = 4 samples each). The protein levels were normalized to control (non-target siRNA). **d** Effects of specific siRNAs against Orais on TG-induced SOCE. *Left panel*, representatives of TG-induced calcium responses recorded in astrocytes transfected with control siRNA or targeting siRNAs against Orais; *right panel*, summary of effects of control siRNA (*n* = 112), Orai1 siRNA (*n* = 76), Orai2 siRNA (*n* = 40), or Orai3 siRNA (*n* = 42). Values represent mean ± SEM, *P < 0.05, compared with control by Student’s *t* test or the one-way ANOVA

To identify which proteins are responsible for the SOCE in spinal astrocytes, we knocked down each of the individual components of the SOCC family using specific siRNAs as demonstrated in our previous study [21]. Astrocytes transfected with the specific STIM1 siRNA at a concentration of 1 μg/ml showed pronounced reduction of STIM1 protein level, while it did not affect STIM2 protein level (Fig. 3a). Similarly, the specific STIM2 siRNA drastically decreased STIM2 protein level but had no effect on STIM1 expression (Fig. 3a). Knockdown of STIM1 greatly decreased TG-induced SOCE, while knockdown of STIM2 significantly reduced the SOCE with less potency (Fig. 3b). Astrocytes transfected with siRNAs targeting Orai1, Orai2, or Orai3 showed a dramatic reduction of their own protein levels at the corresponding bands and showed no interference with each other (Fig. 3c). Knockdown of Orai1 decreased SOCE by 80 %, but knockdown of Orai2 or Orai3 had no effect on SOCE in astrocytes (Fig. 3d). These results demonstrate that STIM1 and Orai1 are primary players responsible for SOCE in spinal astrocytes.

### Depletion of ER Ca^2+^ stores results in STIM1 translocation

**Fig. 4:**
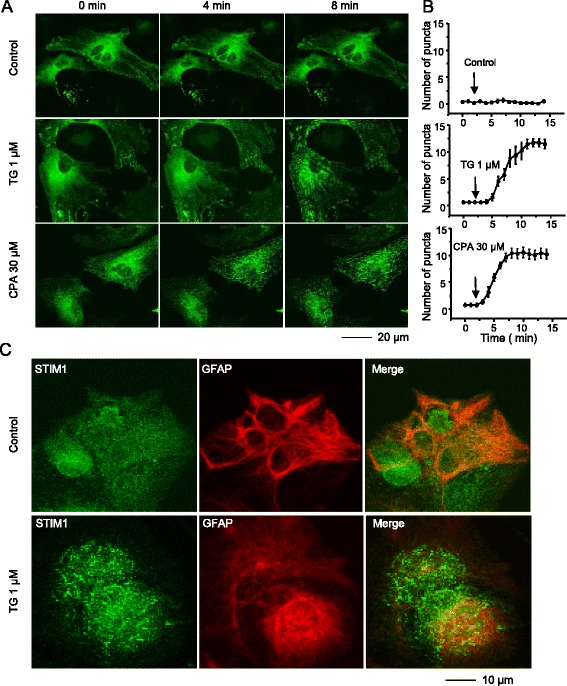
Depletion of calcium stores from endoplasmic reticulum induces STIM1 puncta formation in spinal astrocytes. **a** Live-cell confocal time-lapse images of STIM1-transfected astrocytes treated with 1 μM thapsigargin (TG) or 30 μM cyclopiazonic acid (CPA) at 0, 4, and 8 min. **b** Average number of puncta per 100 μm^2^ induced by respective treatments. Puncta were quantified as spots of high fluorescence intensity ranging from 0.4 to 2.0 μm in diameter size. **c** Confocal images of fixed spinal astrocytes containing endogenous STIM1 puncta after 8 min in the presence and absence of TG (1 μM)

Depletion of Ca^2+^ stores induces STIM1 puncta formation in cell lines [22, 23]. To determine whether this phenomenon also occurs in mouse spinal astrocytes, we transfected astrocytes with STIM1-YFP. Transfection of STIM1-YFP resulted in expression of STIM1 throughout the cell body except the nucleus, and STIM1 randomly formed a few puncta in the resting state. Application of 1 μM TG induced robust STIM1 puncta formation in a time-dependent manner (Fig. 4a). Moreover, the average number of puncta per 100 μm^2^ was increased by TG or CPA treatment (Fig. 4b). To confirm whether TG induces endogenous STIM1 puncta formation in mouse spinal astrocytes, we took advantage of the specific STIM1 antibody. Cells were fixed after application of TG for 8 min. Similar to our transfection data, vehicle treated cells showed few randomly formed puncta, while TG treatment induced obvious puncta formation in GFAP positive cells (Fig. 4c).

### Depletion of ER Ca^2+^ stores increases STIM1 and Orai1 puncta formation

**Fig. 5:**
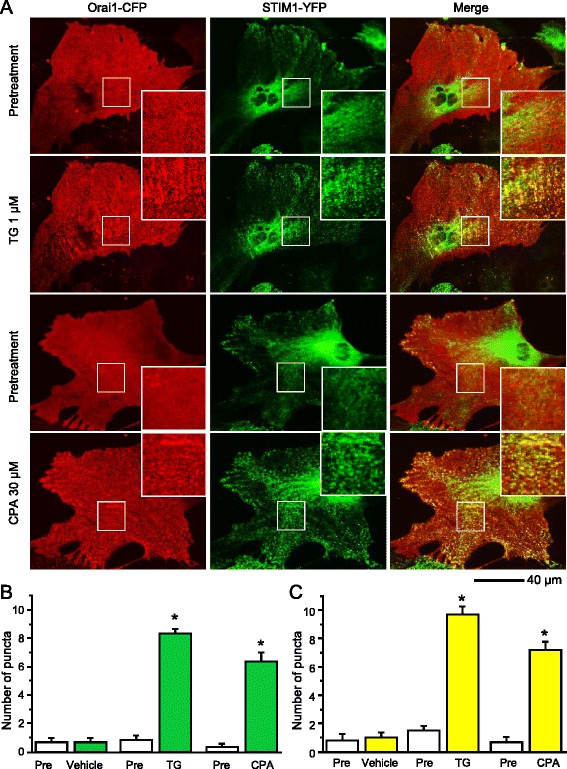
Depletion of calcium stores from endoplasmic reticulum induces STIM1-Orai1 puncta formation in spinal astrocytes. **a** Live-cell confocal images of astrocytes transfected with STIM1-YFP and Orai1-CFP taken before and after 10 min treatment of 1 μM thapsigargin (TG) or 30 μM cyclopiazonic acid (CPA). **b** Average number of STIM1 puncta (*green*) per 100 μm^2^ induced by TG or CPA. **c** Average number of STIM1/Orai1 puncta (*yellow*) per 100 μm^2^ induced by TG or CPA. Puncta were quantified as spots of high fluorescence intensity ranging from 0.4 to 2.0 μm in diameter size before and after 10 min TG and CPA treatments. Values represent mean ± SEM, *P < 0.05, compared with pretreatment by Student’s *t* test

To examine whether STIM1 forms puncta with Orai1 in astrocytes, Orai1-tagged with cyan fluorescent protein (Orai1-CFP) and STIM1-YFP were co-transfected in astrocytes. In untreated cells, Orai1-CFP was evenly expressed throughout the cell, except for the nucleus. Following the addition of TG and CPA, Orai1-CFP showed a marked change in distribution and puncta-like formations were seen throughout the astrocytes. TG or CPA triggered both STIM1 and Orai1 reorganization, leading to a pronounced enhancement of STIM1–Orai1 co-localization (Fig. 5). These results suggest that STIM1 interacts with Orai1 upon Ca^2+^ store depletion. This result further supports the notion that depletion of ER Ca^2+^ stores causes STIM1 translocation leading to activation of Orai1 and the subsequent calcium influx into astrocytes.

### SOCE regulates cytokine production in spinal astrocytes

**Fig. 6:**
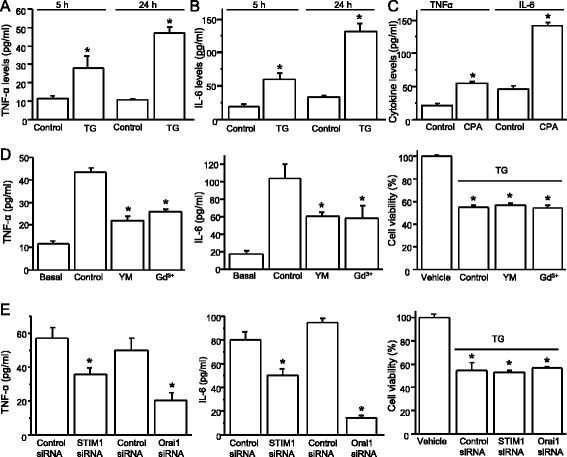
Depletion of Ca^2+^ store induces cytokine production in spinal astrocytes. **a** TG-induced TNF-α production. **b** TG-induced IL-6 production. **c** CPA-induced TNF-α and IL-6 production (*n* = 6–8). **d** Effects of YM-58483 (YM, 3 μM) and GdCl_3_ (Gd^3+^, 0.3 µM) on TG-induced cytokine production and reduction of cell viability (*n* = 6–7). **e** Effects of knockdown STIM1 or Orai1 on TG-induced cytokine production and reduction of cell viability (*n* = 6). Values represent mean ± SEM, *P < 0.05, compared with control by Student’s *t* test or the one-way ANOVA

Ca^2+^ signals in astrocytes mediate a remarkable variety of cellular functions including cytokine production. To determine whether SOCE plays a role in cytokine production from spinal astrocytes, we tested effects of TG and CPA on cytokine production. Astrocytes were pretreated with 1 μM TG in the Ca^2+^-free Tyrode’s solution for 5 min and then replaced with MEM culture medium containing TG for 5 or 24 h. TNF-α production was significantly increased after a 5-h treatment compared to control cells, and a more robust TNF-α production was observed at the 24-h time point (Fig. 6a). Similarly, TG-induced IL-6 production was greater after a 24-h treatment (Fig. 6b). To further determine that activation of SOCE results in cytokine production, we tested the effect of CPA on cytokine production in spinal astrocytes using the same conditions. CPA also significantly increased production of TNF-α and IL-6 after a 24-h treatment (Fig. 6c). To confirm the involvement of the SOCE in pro-inflammatory cytokine production, we investigated the effect of SOCE inhibition on TG-induced cytokine production. Astrocytes were pretreated with SOCC inhibitors 1 μM YM-58483 and 0.3 μM GdCl_3_ for 15 min. Both inhibitors significantly reduced TG-induced TNF-α and IL-6 production (Fig. 6d). These results suggest that SOCE plays a role in cytokine production.

TG has been implicated as a potent inductor of ER stress and apoptotic cell death [24, 25]. To determine whether SOCCs contribute TG- induced reduction of cell viability, an MTT assay was performed in TG-treated astrocytes after 5 and 24 h. At the earlier time point, TG had no effect on cell viability despite the significant increase in both TNF-α and IL-6 production. Conversely, TG significantly reduced cell viability to 55 % after 24 h, while YM and GdCl_3_ had no effect on TG-induced reduction of cell viability (Fig. 6d). These data suggest that SOCCs are involved in TG- and CPA-induced cytokine production but not in TG-induced decrease in cell viability. To further confirm that SOCC proteins STIM1 and Orai1 play a role in cytokine production, astrocytes were transfected with control siRNA or targeting siRNAs. We found that knockdown of STIM1 or Orai1 significantly decreased the cytokine production but had no effect on TG-induced reduction of cell viability (Fig. 6e). These results demonstrate that STIM1 and Orai1 play a role in pro-inflammatory cytokine production in spinal astrocytes.

### Orai1 and STIM2 are involved in LPS-induced cytokine production

**Fig. 7:**
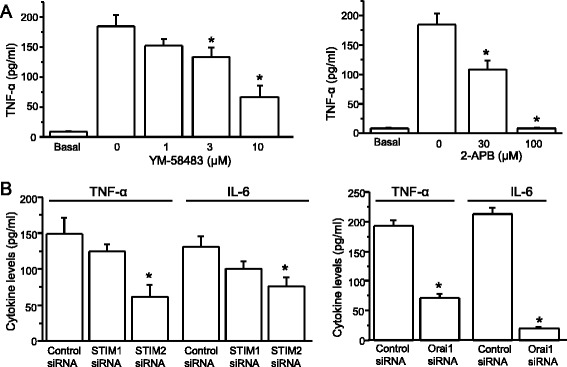
LPS-induced cytokine production is inhibited by SOC inhibitors or knockdown of STIM2 or Orai1. **a** Effects of YM-58483 and 2-APB on LPS-induced TNF-α and IL-6 production. **b** Effects of knockdown STIM1, STIM2, or Orai1 on LPS-induced TNF-α and IL-6 production. *n* = 6. Values represent mean ± SEM, *P < 0.05, compared with control by the one-way ANOVA or Student’s *t* test

The findings described above prompted us to test whether SOCCs are involved in cytokine production induced by LPS, which is a well-established stimulator of cytokine production in astrocytes. Addition of 2 ng/ml LPS largely increased IL-6 and TNF-α after treatment for 5 h (Fig. 7a). We then tested the effects of SOCC inhibitors 2-APB and YM-58483 on LPS-induced IL-6 and TNF-α production, both of which significantly reduced LPS-induced IL-6 and TNF-α production in a concentration-dependent manner (Fig. 7a) and did not affect cell viability (data not shown). These results suggest that SOCCs may be involved in LPS-induced cytokine production from spinal astrocytes. To confirm that SOCCs play a role in LPS-induced cytokine production, we transfected astrocytes with the specific siRNA to knock down STIM1 in spinal astrocytes. Interestingly, knockdown of STIM1 had no significant effect (Fig. 7b). We then tested whether STIM2 is involved in LPS-induced cytokine production. Indeed, reduction of STIM2 by its specific siRNA significantly attenuated LPS-induced IL-6 and TNF-α production, but did not significantly affect cell viability (93 ± 7 % in the STIM2 siRNA group vs 100 ± 5 % in the control siRNA group). Finally, we examined whether Orai1 has an effect on cytokine production induced by LPS, Surprisingly, decrease in Orai1 expression by its siRNA drastically reduced TNF-α production and almost abolished IL-6 production (Fig. 7b), while it had no effect on cell viability (104 ± 4 %) compared to control siRNA. These results indicate that STIM2 and Orai1 play a critical role in LPS-induced cytokine production.

## Discussion

In the present study, we demonstrated that SOCCs are expressed and functional in spinal astrocytes. We identified STIM1, STIM2, and Orai1 as players responsible for SOCE in spinal astrocytes. Our data revealed that STIMs and Orai1 play a role in TG- and LPS-induced cytokine production. These findings indicate the functional importance of STIM1, STIM2, and Orai1 in spinal astrocytes.

Astrocytes are the predominant cell type responsible for the maintenance of brain homeostasis in the CNS. SOCE is a widely recognized calcium signal in non-excitable cells including rat brain astrocytes [26]. However, its molecular identity remains controversial. A recent study has shown that STIM1 and Orai1 mediate SOCE in rat cortical astrocytes [18]. SOCC proteins and SOCE have not been reported in spinal cord astrocytes. In this study, we demonstrated that the members of the SOCC family are differentially expressed in spinal astrocytes. The calcium imaging data showed that depletion of ER calcium stores with TG or CPA-induced robust calcium entry in astrocytes, which is inhibited by SOCC inhibitors 2-APB, GdCl_3_, and YM-58483. We further identified the components of SOCCs responsible for SOCE using specific siRNAs that target individual members of the SOCC family. We found that knockdown of STIM1 dramatically decreased TG-induced SOCE. Although STIM1 has been shown to play a crucial role in the store-operated coupling process, the role of STIM2 remained unclear. Some reports showed that STIM2 has no effect or an inhibitory effect on SOCC activation [27, 28], but our current study, as well as others, have indicated that STIM2 also acts as a Ca^2+^ sensor [21, 23, 29, 30]. Here, we also demonstrated that STIM2 is a significant player of SOCE in spinal astrocytes, though it is not as strong as STIM1. Similar to other cell types [31, 32], Orai2 and Orai3 are not responsible for the SOCE in spinal astrocytes. Knockdown of Orai1, however, reduced SOCE by 80 %, suggesting that Orai1 might be the only STIM1-activated channel involved in TG-induced SOCE. Thus, we revealed for the first time that STIM1, STIM2, and Orai1 are the molecular components responsible for SOCE in spinal astrocytes.

It is well documented that a decrease in ER Ca^2+^ results in a profound intracellular redistribution of STIM1 from a uniform ER pattern to spatially discrete areas termed as puncta. This leads to the activation of the Orai1 channel at the plasma membrane, allowing Ca^2+^ entry from the extracellular milieu [33–35]. In this study, we also examined STIM1 redistribution and puncta formation with Orai1. Our live-cell confocal images clearly showed that reduction of ER Ca^2+^ by TG or CPA-induced robust STIM1 puncta formation. Interestingly, we also observed that TG and CPA induced Orai1 puncta-like formation. Under the resting state, STIM1 formed a few puncta with Orai1. After TG and CPA treatments, STIM1 formed substantial puncta that co-localized with Orai1. Our result is consistent with the previous findings that Orai1 and STIM1 move in a coordinated fashion to form clusters in plasma membrane [36].

Pro-inflammatory cytokine production is one of the biological markers of astrocyte activation. Numerous studies reported that pro-inflammatory cytokines like TNF-α and IL-6 are involved in pain hypersensitivity [37, 38]. They are up-regulated in pain conditions [39, 40], and intrathecal injection of these pro-inflammatory cytokines can directly induce pain responses [41, 42]. Unlike the well-known critical role of SOCCs in cytokine production in immune cells [10], the role of SOCCs in cytokine production in astrocytes has not been elucidated. In the present study, we discovered that activation of SOCCs by TG or CPA could induce TNF-α and IL-6 production in spinal astrocytes. Both Gd^3+^ and YM-58483 significantly decreased the TNF-α and IL-6 production induced by TG. More importantly, we found that knockdown of STIM1 and Orai1 decreased TNF-α and IL-6 production in spinal astrocytes. Consistent with previous studies, we observed that TG reduced cell viability. However, this reduction was not mediated by SOCE since inhibition of SOCE or knockdown of STIM1 and Orai1 did not affect TG-induced reduction of cell viability.

Since inhibition of Ca^2+^-ATPase is not a physiological or pathological approach, we stimulated astrocytes using LPS, a well-defined toll-like receptor (TLR) 2/4 agonist. TLR2/4 receptors are implicated in several pathological conditions such as rheumatoid arthritis [43, 44]. LPS induced a large amount of TNF-α and IL-6 production which was blocked by SOC inhibitors and knockdown of STIM2 and Orai1 but not by knockdown of STIM1. These findings suggest that STIM2 and Orai1 contribute to LPS-induced cytokine production. LPS has been shown to induce Ca^2+^ release from Ca^2+^ stores and elicit Ca^2+^ oscillations in murine macrophages [45, 46]. Previous studies have demonstrated that STIM2 can activate Ca^2+^ oscillations upon smaller decreases in ER Ca^2+^ by low-level agonist concentrations [30, 47]. We used a very low concentration (2 ng/ml) of LPS, which may only activate STIM2. This might explain why a decrease in STIM2 can reduce LPS-induced cytokine production. Another possibility is that STIM2 constitutively interacts with Orai1 to regulate basal Ca^2+^ concentration, which enhances transcriptional activity [48, 49]. However, the exact role of STIM2 and Orai1 in LPS-induced production of pro-inflammatory cytokines and the cross-talk between the STIM2/Orai1 and TLR signaling remain to be explored.

## Conclusions

We demonstrated that SOCCs are expressed in spinal astrocytes, and we identified STIM1, STIM2, and Orai1 as the components responsible for SOCE. Our data also demonstrated that SOCE can induce cytokine production and that STIM2 and Orai1 play an important role in LPS-induced cytokine production in spinal astrocytes. These findings reveal a new calcium signal in spinal astrocytes and may suggest a possible therapeutic target for treatment of chronic pain and other CNS disorders associated with abnormal astrocyte activities.

## Abbreviations

2-ABP, 2-aminoethyldiphenyl borate; CNS, central nervous system; CPA, cyclopiazonic acid; DMEM, Dulbecco’s modified Eagle’s; ELISA, enzyme-linked immunosorbent assay; ER, endoplasmic reticulum; FCS, fetal calf serum; GFAP, glial fibrillary acidic protein; HBSS, Hank's Balanced Salt Solution; IL-6, Interleukin-6; LPS, lipopolysaccharide; MEM, medium minimum essential media; siRNA, small inhibitory RNA; SOCCs, store-operated calcium channels; SOCE, store-operated Ca^2+^ entry; STIM, stromal cell-interaction molecule; TG, thapsigargin; TLR, toll-like receptor; TNF-α, tumor necrosis factor-α; YM-58483, N-[4-[3,5-Bis(trifluoromethyl)-1H-pyrazol-1-yl]phenyl]-4-methyl-1,2,3-thiadiazole-5-carboxamide
